# The Social Context of Malevolent Creativity

**DOI:** 10.3390/bs15050630

**Published:** 2025-05-05

**Authors:** Harun Tadik, A. Kadir Bahar, Mark A. Runco

**Affiliations:** 1 Department of Educational Psychology, University of Georgia, Athens, GA 30602, USA; 2 Creativity Research & Programming, Southern Oregon University, Ashland, OR 97520, USA; runcom@sou.edu; 3 Radical Creativity, Aalto University, 02150 Espoo, Finland

**Keywords:** malevolence, benevolence, negative creativity, morality, dark side of creativity

## Abstract

The purpose of the present study was to investigate the influence of social context and morality on malevolent creativity. The sample consisted of 217 (176 female, 41 male) undergraduate and graduate students in a Southeastern public research university in the U.S. Six different open-ended socially oriented problems (three Divergent Thinking (DT) Social Games and three DT Realistic Presented Problems tasks) were used to explore the way malevolent creativity differs in two sets of problem tasks. Realistic Presented Problems are constructed differently from Social Games tasks in such a way that they include unfair, disturbing, and threatening contextual factors, while Social Games problems have more neutral and everyday problem scenarios. Participants also completed the Self-Report of Creative Traits and morality measures. The findings of the study indicated that fluency and originality were related significantly to malevolent creativity. Further, social contexts in DT tasks led to a significant difference in malevolent creativity. Participants generated significantly more malevolent creative responses in Realistic Presented Problems than in Social Games tasks. The results also revealed that morality was significantly correlated with creative personality, but the results provided no evidence that morality moderates the relation between creativity indices and malevolent creativity.

## 1. Introduction

There is increasing interest in malevolent creativity within creativity research, sparking ongoing debate about whether creativity inherently possesses a dark side. Generally, creativity is perceived as a positive and valuable personal ability. Runco (2010) suggested that creativity itself is not inherently dark. Instead, he argued that the creative process and creative products should be considered separately, implying that malevolent creativity is more closely related to creative products and is independent of the creative process. Conversely, several studies have indicated that creativity may indeed have a dark side that leads to undesirable outcomes (D. H. Cropley et al., 2008; Gino & Ariely, 2012; McLaren, 1993; Niepel et al., 2015; Walczyk et al., 2008). Creativity requires novelty and usefulness for a specific purpose or to solve a problem (D. H. Cropley et al., 2008), and it is controversial whether the dark side of creativity can be considered useful or appropriate. Novelty is evident in many criminal behaviors, competitions, and historical leaders who have caused significant pain, death, and damage worldwide (D. H. Cropley et al., 2008; Sternberg, 2010).

McLaren (1993) provided numerous examples of how the dark side of creativity manifests across different fields, such as the arts, science, and technology. In a later study, James et al. (1999) conceptualized creativity into two categories: positive and negative. They defined negative creativity as “the intent to harm, hinder, harass, destroy, or achieve unfair or undeserved advantage” (pp. 212–213). D. H. Cropley et al. (2008) distinguished between benevolent and malevolent creativity, where the former refers to using creativity for personal or group benefit, and the latter involves using creativity intentionally to harm others. They highlighted that individuals, groups, or entire societies can use their creative potential intentionally to achieve goals, despite the serious adverse consequences for others. Those who engage in malevolent creativity are aware of the potential adverse or even dangerous outcomes for others but continue to use creativity in a negative way deliberately (A. J. Cropley, 2010; D. H. Cropley et al., 2008).

The relationship between creativity and its dark side has been investigated in several empirical studies (Gino & Ariely, 2012; Niepel et al., 2015; Walczyk et al., 2008). Walczyk et al. (2008) conducted a study using social dilemma scenarios to explore the link between creativity and lying. Their results indicated that creativity in lying is associated with divergent thinking and ideation, finding a positive correlation between the number of lies and levels of divergent thinking and ideation. Gino and Ariely (2012) conducted a series of studies demonstrating that individuals with creative potential and a creative mindset are more likely to engage in dishonest behaviors because they find it easier to justify and rationalize these actions. Beaussart et al. (2013) also suggested that integrity negatively correlates with creativity. However, a more recent study by Niepel et al. (2015) found that creativity does not negatively influence honesty, particularly in ethical decision-making. Although research on this topic is limited, the cited literature supports the notion that creativity may have a dark side.

It is important to note the nuances in defining the dark side of creativity, as malevolent and negative creativity differ slightly. As previously discussed, malevolent creativity refers to intentional harm, while negative creativity may involve unintentional adverse consequences of otherwise positive creative efforts (James & Drown, 2008). Since the present study focuses on intentional changes in creative behavior, the term “malevolent creativity” is used to describe the deliberate use of creative potential for harmful purposes.

### 1.1. Social Context and Malevolent Creativity

It is unsurprising that individuals may adjust their behaviors when exposed to different social contexts, and malevolent creativity is not exempt from the influence of context. Social context can be a significant factor that encourages individuals to use their creative potential for malevolent purposes (Baas et al., 2019; Harris & Reiter-Palmon, 2015; Kapoor & Khan, 2019; Walczyk et al., 2008). Individuals may respond to socially oriented problems based on contextual characteristics and make mindful choices about whether to engage in malevolently creative behaviors or ideation. Intentionality is a crucial aspect of malevolent creativity (A. J. Cropley, 2010; D. H. Cropley et al., 2008; Gruber, 1993; James et al., 1999; Runco, 1993), and it is expected that the surrounding social environment influences mindful decisions regarding creative behaviors and ideas (Amabile, 2018; Perry-Smith & Shalley, 2003; Simonton, 2000). Individuals may choose to use malevolent creativity in specific jobs or tasks (Gino & Ariely, 2012) and may attempt to deceive depending on the social context (Walczyk et al., 2008). In conceptualizing positive versus negative creativity, James et al. (1999) suggested that environmental and individual factors interact with one’s emotional state, and depending on the input, creative potential can produce various adverse outcomes. Among the environmental factors, they cited negative climate, social complexity, culture, perceived unfairness, and diversity as factors that trigger negative emotions, leading individuals to set negative goals and utilize creative potential to achieve these adverse objectives.

In an earlier empirical study, Clark and James (1999) examined how perceived justice influences malevolent creativity. They asked a group of college students to alphabetize a list of supposed applicants within a given time and informed participants they could earn up to six lottery tickets for a USD 50 drawing based on their performance. They fulfilled their promise for half of the sample, giving each participant six tickets, while the unjust condition group received no tickets. Regardless of the justice condition, all participants then responded to two divergent thinking tasks, constructed as positively and negatively socially oriented problems. The study’s results revealed that those treated unfairly generated more malevolent creative ideas than those in the fair condition, supporting the argument that specific contextual factors effectively provoke malevolent creativity.

Explaining malevolent creativity, Harris and Reiter-Palmon (2015) suggested that interactions among three main factors—implicit aggression, premeditation, and provoking situations—are effective in generating malevolently creative ideas. Harris and Reiter-Palmon noted that some individuals have greater implicit aggression and consider their actions less. Their empirical study on college students supported their hypothesis, revealing that those with high implicit aggression and low premeditation are likely to express malevolent creativity in socially oriented problems that provoke malevolent use of creativity. These findings indicate that personality and social context should be considered to understand malevolently creative behaviors.

In another study, Harris et al. (2013) found that the type of socially oriented problem is a significant predictor of malevolent creativity. They used three different tasks with varying social context scenarios, one of which had a relatively more uncomfortable social context, deemed to elicit greater malevolent creativity. Their study demonstrated that differences in social context effectively manipulated malevolent creativity. From a different perspective, Kapoor and Khan (2019) compared malevolent creativity in real-life and object-based problems, commonly known as Alternative Uses tasks. They suggested that real-life problem tasks elicited more malevolent responses than object-based tasks. Baas et al.’s (2019) study manipulated social threat, creating two groups of participants exposed to high versus low social threat conditions. Similarly, they indicated that participants in the high social threat condition were more malevolently creative. Other studies in the field of criminal justice have also shown that social context is an important factor in violence (Fuchs, 2008), bullying (Espelage et al., 2000), and crime in general (Miethe & Meier, 1994).

### 1.2. Creativity and Morality

The relationship between creativity and morality is crucial for understanding malevolent creativity as most, if not all, malevolently creative ideas and actions contradict moral values and expectations. In his seminal theory of moral development, Kohlberg (1984) posited that “conventionality” is an essential concept that determines the stages of moral development. According to Kohlberg, when children are socialized to behave conventionally, they are more likely to exhibit conventional behaviors and values. However, a commonly held assumption in the creativity literature suggests a negative relationship between creativity and conventionality, implying that creativity and morality are mutually exclusive (Runco, 1993; Shen et al., 2019). Morality and creativity appear contradictory, as individuals with high moral standards are expected to follow traditions and rules, whereas creativity is associated with deviating from traditions, breaking rules, and expressing unexpected and novel ideas and behaviors (Runco, 2009, 2014). As documented in the malevolent creativity literature, creativity is linked to various negative personality traits (D. H. Cropley et al., 2013). For instance, empirical studies have revealed that creative individuals are more likely to fabricate lies (Walczyk et al., 2008), express hostility (Feist, 1998), and demonstrate dishonesty (Beaussart et al., 2013).

Conversely, the creativity literature also presents an alternative view suggesting that creativity is compatible with morality. In his review, Runco (1993) concluded that conventionality was not a useful framework for understanding creativity in the moral domain, as numerous social and moral issues could benefit from creative solutions (Gruber, 1985, 1993; Runco, 1993, 2009; Runco & Nemiro, 2003). Other empirical studies have demonstrated a positive correlation between creativity and morality or morality-relevant concepts (Chen & Hou, 2016; Liu et al., 2014; Yurtsever, 1998). From a theoretical perspective, Campbell (1960) proposed that creative ideation is a two-stage process consisting of blind ideation followed by selective retention. Given that the ideation stage is blind, it is conceivable that it is free from moral standards, with morality likely intervening during the selection or creative production stage. Consequently, a variety of ideas, both moral and immoral, could be generated during the ideation stage. Individuals can then intentionally choose to exhibit certain moral behaviors under different conditions or decide to channel their creative potential toward developing creative solutions for moral issues.

### 1.3. The Present Study

The purpose of the present study was to investigate the effect of social context on malevolent creativity and to explore how morality moderates the relationship between creative potential and malevolent creativity, as well as between creative personality and malevolent creativity. It is unsurprising that individuals who frequently exhibit hostility and aggression are likely to generate and express malevolent creativity (Hao et al., 2016; Harris & Reiter-Palmon, 2015). Notorious figures such as dictators, serial killers, robbers, and terrorists often employ novel and surprising ideas to achieve their ambitions. However, malevolent creativity is not confined to a small group of criminals; it can manifest in everyday life through actions like lying, deception, or cheating (James et al., 1999; Harris & Reiter-Palmon, 2015; Walczyk et al., 2008). While it is less common for ordinary individuals to practice malevolent creativity, it can arise when they encounter provoking social contexts (Baas et al., 2019; Harris & Reiter-Palmon, 2015). Therefore, ordinary people may express extraordinary ideas or behaviors when certain social contexts prompt the use of creativity for aggressive purposes.

In this study, we examined the influence of social context on the variation in malevolent creativity across different socially oriented problems, specifically comparing disturbing and unfair contexts to normal, non-disturbing daily contexts. The present study distinguishes itself from previous research by focusing on how the same individuals engage in malevolent creativity when experiencing disturbing versus non-disturbing social context scenarios, rather than comparing separate groups exposed to different contextual problems.

Furthermore, we explored the relationship between malevolent creativity, creative potential, creative personality, and morality. Although there is a growing body of literature on creativity and morality-related concepts, such as religiosity, integrity, and ethical decision-making, the association between malevolent creativity and morality remains understudied in creativity research. Given the mixed results regarding the association between creativity and morality, integrating and exploring morality in the context of malevolent creativity research could enhance our understanding of the underlying factors of malevolent creativity and open new avenues for future studies in the field of the dark side of creativity. Another objective of this study was to examine the moderating role of morality in the relationship between creative personality and creative potential with malevolent creativity. Specifically, the study aimed to address the following research questions:

1. What is the relationship between morality, malevolent creativity, creative potential, and creative personality?
2. Is there a significant difference in malevolent creativity based on variations in social context?
3. How does morality moderate the relation between creative potential and malevolent creativity, as well as creative personality and malevolent creativity?

## 2. Method

### 2.1. Sample

The sample comprised 217 undergraduate and graduate students (176 female, 41 male) predominantly majoring in education and social sciences at a Southeastern public research university. Participants’ ages ranged from 17 to 49 years, with a mean age of 21.81 (*SD* = 5.47). The racial composition of the sample was as follows: 69% White, 12% Black, 8% Asian/Pacific Islander, 6.5% Hispanic, and 9% from other racial backgrounds.

### 2.2. Procedures

Data were collected using Qualtrics, an online survey platform designed for research purposes. The study link was disseminated through the SONA system—an online research pool platform that offers credit for research participation—and shared with professors who agreed to distribute it to their students. Participants received partial course credit as an incentive for their participation. Regarding the efficacy of online data collection in creativity, it is important to recognize that online assessment of creativity yields similar outcomes comparable to in-person methods (Hass, 2015). The consent form for participation was distributed to all participants and signed electronically. Upon providing informed consent, participants were requested to provide their demographic information, including age, gender, and educational level. Subsequently, they completed the following tasks in order: Realistic Presented Problems (RPP); the Creative Activity and Accomplishment Checklist (CAAC), focusing on the morality domain; Divergent Thinking Social Games; and Self-Report of Creative Traits (SRCT), a self-report measure assessing creative personality.

All measures, including the demographic survey, were completed in a single session without real-time guidance or monitoring for participants while completing the online tasks. However, a detailed instruction was provided for each measure. Participants with incomplete measures were excluded from the study sample. Each divergent thinking task was allocated a time limit of 3 min, and two raters with expertise in creativity research evaluated the participants’ responses.

### 2.3. Measures

#### 2.3.1. Measure of Malevolent Creativity—Set I: Divergent Thinking (DT) Realistic Presented Problems (RPP)

The Realistic Presented Problems (RPP) consists of three tasks designed to elicit solutions to potential real-life problems, specifically incorporating unfair and disturbing social context scenarios to reveal the darker side of participants’ creativity. The test was adapted from Runco and Okuda (1988). One scenario is directly derived from Runco and Okuda’s original RPP, the second is adapted from a common workplace ethical dilemma (retrieved from http://mallenbaker.net/ and accessed on 12 January 2021), and the third is based on Harris et al.’s (2013) study. Prior to presenting the problems, participants received the following instructions:

*In the next few sections, we will describe a few problems that may occur at school and work. Your task is to first read about the problem and then try to write down **as many solutions as you can** for each one. You will have 3 min for each case. Use your time wisely and list as many alternatives as you can.*

The tasks addressed a classroom problem, a managerial issue in a workplace setting, and a scenario involving Sally. In the classroom task, participants were asked to assume they are in a class where their friend Pat, sitting next to them, is being disruptive during a class they are eager to learn from. For the managerial task, participants were instructed to “*Imagine that you are working in a company and have been placed under someone who is very political and actively hostile to you. It is obvious that the manager is misrepresenting you up the chain, making it look like you are not performing well. It may only be a matter of time before the manager successfully tarnishes your reputation in the company and eventually forces you out*”. The final task involved Sally, a new college student assigned a roommate who plays loud music while Sally tries to study. Despite her timidity, Sally fears the situation will escalate if not addressed. Participants were asked to propose what they would do or say in each scenario.

#### 2.3.2. Measure of Malevolent Creativity—Set II and Creative Potential: Divergent Thinking (DT) Social Games (SG)

The Social Games (SG) tasks were incorporated to explore how the dark side of creativity is utilized in socially and emotionally less disturbing everyday contexts. The test comprised three tasks, each requiring participants to generate multiple expressions of a target idea. Prior to presenting the tasks, participants received the following instructions, accompanied by an example to clarify expectations:

*Sometimes we need to find alternative ways to say things to other people. You will be given a blunt expression (“you have body odor”) and asked to list different ways of conveying that idea to someone. The goal is to list as many different ways as you can to convey the target idea. Rather than saying, “You have body odor,” you may say, “Do you smell something?” or “Have you been working out?” There are many ways of expressing the target ideas. You will have 3 min for each task, and the more you list, the better!*

The tasks included the following prompts: *(1) List as many ways as you can to convey the idea that the meal your friend has just prepared for you does not taste good. In fact, it tastes bad! (2) List different ways of conveying to someone that they are not dressed appropriately. (3) List as many ways as you can to convey the idea that someone’s performance (say in a sport or on a test) was very poor.*

It is important to note that while the RPP and SG tests both focus on socially oriented problems, they differ in approach. The SG tasks originally encouraged participants to convey ideas politely. However, to avoid prompting malevolent responses, this aspect was removed from the instructions, and more socially neutral guidance was provided. As discussed, RPP involves personally disturbing social context scenarios, whereas SG tasks are relatively non-disturbing and are not intended to provoke aggressive responses. Consequently, RPP will be referred to as the “disturbing” group and SG as the “non-disturbing” group of Divergent Thinking (DT) tests to prevent any potential confusion. Additionally, explicit instructions requesting creative responses were avoided. Instead, participants were simply asked to generate as many responses as possible within the given time. Given that creative responses are typically novel and deviate from expected behaviors, explicitly asking for creative responses could inadvertently amplify malevolent responses. Therefore, standard instructions were employed that neither encouraged nor discouraged benevolent or malevolent responses, focusing solely on the generation of responses within the context scenarios.

#### 2.3.3. Measure of Morality: The Creative Activities and Accomplishment Checklist (CAAC)-Morality Scale

The Creative Activities and Accomplishment Checklist (CAAC) morality domain scale, part of the Runco Creativity Assessment Battery, was utilized to measure creative activities within the morality domain. High scores on this measure indicate a high level of engagement in moral activities. The subtest comprises 20 items and has demonstrated good internal consistency, with a reliability coefficient of 0.85. Sample items from the subtest are as follows: “Told the truth even though it may have negative consequences”, “Posted about morality, justice, equality, or similar issues in your social media accounts?”, and “Volunteered your time to a charity”. Participants responded to each item using a 4-point Likert scale with the following options: Never, Once or twice, 3–5 times, and More than 5 times. It is important to note that, as seen in the sample items, the scale captures a wide variety of proactive moral behaviors and activities.

#### 2.3.4. Measure of Creative Personality: The Self-Report of Creative Traits (SRCT)

The Self-Report of Creative Traits (SRCT) is a self-assessment measure of creative personality encompassing 12 distinct traits (Runco et al., 2016, 2017). It was initially titled “How Would You Describe Yourself?” (HWYDY) in the 2016 study. Each item on the SRCT targets a specific trait and provides a brief description, such as “Spontaneous: You live in the moment and don’t try to be someone else. You are yourself”. Participants were asked to self-report the frequency or extent to which they exhibit each trait using a 6-point Likert scale ranging from “never” to “always”. To mitigate response bias, four of the traits were contraindicative and were reverse-coded for scoring purposes.

#### 2.3.5. Scoring Procedures for Divergent Thinking Tests

The responses to both Divergent Thinking (DT) tests were evaluated by the first author and an external rater with expertise in creativity research and training in scoring creativity tests. The author doing the scoring was blind, in that he did not have subject, group, or condition identifiers. The second scorer was entirely blind about the objectives of the study, as well as identifiers, tasks, and conditions. For scoring malevolent creativity, the procedure outlined by Harris et al. (2013) was employed. Originality was assessed based on the responses’ frequency, structure, and use of humor and imagination. Malevolent creativity was determined by the harmful nature of the responses; any response likely to inflict physical, psychological, mental, or emotional harm on any party or member of the social context was classified as malevolent. A positive solution was defined as a response expressing thoughts and feelings with no risk of harm to oneself or others in the social context.

The scoring process involved three steps to enhance inter-rater agreement. Initially, responses were categorized as either positive or negative. Subsequently, each response was rated for both originality and negativity on a 6-point scale (1 = very unoriginal to 6 = very original; 1 = very negative to 6 = very positive). It is noteworthy that the 6-point scale was utilized to improve the inter-rater reliability and to determine whether a response should be considered negative and original. Corresponding scale numbers were not used for further analyses, as they were solely instrumental in determining the categories. Finally, the raters revisited responses on which they disagreed to reach a consensus. To qualify as malevolent creativity, a response had to be both negative and original. For instance, if a participant provided five responses for a particular test and only one of those responses met both criteria, the participant received a single malevolent creativity score. The total number of malevolently creative responses for each participant constituted their malevolent creativity score. The inter-rater reliability coefficient for the six DT tasks assessing malevolent creativity ranged from 0.72 to 0.90.

The DT Social Game tasks were also scored by the same raters to assess creative potential, with scores for fluency and originality calculated for each participant. For originality assessment, a procedure considering response frequency was used; any response given by 3% of the sample or fewer was deemed original. Participants’ number of responses was used to calculate fluency. The inter-rater reliability for originality was 0.76.

### 2.4. Data Analysis

Different statistical analyses were employed to address the research questions. Initially, reliability analyses were undertaken to evaluate the consistency of all measures utilized in the study. To examine the correlations among malevolent creativity, creative potential, creative personality, and morality, zero-order correlations were performed. Subsequently, the variation in malevolent creativity across different social contexts was assessed using paired sample *t*-tests. This helped determine how malevolent creativity manifested differently in these contexts. Lastly, to address the final research question, multiple regression moderation analyses were conducted. Here, malevolent creativity served as the dependent variable, while creative potential and personality acted as predictors, with morality functioning as the moderator. This moderation analysis aimed to explore whether the relationship between creative personality and creative potential concerning malevolent creativity varied based on levels of morality (Cohen & Cohen, 1983).

## 3. Results

Table 1 presents the descriptive statistics for morality, SCRT, fluency, originality, and malevolent creativity scores across each task, alongside the total malevolent creativity scores for three DT Social Games tasks and three DT Realistic Presented Problems tasks. The reliability coefficients were found to be 0.85 for CAAC-Morality, 0.75 for SRCT, 0.82 for DT Social Games fluency, 0.85 for Realistic Presented Problems fluency, and 0.65 for DT Social Games originality.

### 3.1. Correlation Analysis

To address the first research question, we examined the correlations between malevolent creativity, morality, creative personality, and creative potential indices, specifically fluency and originality, as reported in Table 2. The findings revealed significant correlations: morality was linked with creative personality (SRCT) (*r* = 0.34, *p* < 0.001) and fluency (*r* = 0.16, *p* = 0.017). Furthermore, malevolent creativity was significantly associated with both fluency (*r* = 0.42, *p* < 0.001) and originality (*r* = 0.49, *p* < 0.001).

### 3.2. Mean Comparison of Malevolent Creativity Based on Social Context Differences

A paired sample *t*-test was conducted to determine whether there is a sizeable difference between malevolent creativity scores for different social contexts. The results indicated a significant difference between SG (*M* = 0.77, *SD* = 1.46) and RPP (*M* = 1.61, *SD* = 2.50) contexts (*t*(216) = −5.78, *p* < 0.001, *d* = −0.412). Participants exhibited more malevolently creative responses in RPP tasks, characterized by personally unfair, disturbing scenarios compared to SG tasks, which involved more everyday scenarios.

A repeated measure ANOVA was employed to delve deeper into differences among the DT tasks. Mauchly’s Test of Sphericity indicated that the assumption of sphericity was violated (*χ*^2^(14) = 68.34, *p* < 0.01); thus, a Greenhouse–Geisser correction was used. The results indicated a significant difference between the socially oriented DT tasks (*F* (4.47, 925.67) = 13.81, *p* < 0.001). Post hoc analyses with Bonferroni correction revealed that the average number of malevolently creativity responses for each Sally (*M* = 0.46, *SD* = 0.98), Classroom (*M* = 0.51, *SD* = 0.98), and Manager (*M* = 0.65, *SD* = 1.14) task was significantly higher than for Performance (*M* = 0.16, *SD* = 0.69) and Dress (*M* = 0.20, *SD* = 0.53) tasks. There was no statistically significant difference between the Taste (*M* = 0.42, *SD* = 0.86) task and any of the Sally, Classroom, and Manager tasks. The Taste task elicited significantly more malevolent creativity responses among DT Social Game tasks, though no significant differences were observed in DT Realistic Presented Problems. These findings suggest that social context may play a pivotal role in motivating malevolent creativity.

### 3.3. Predictors of Malevolent Creativity

We conducted multiple regression analyses to explore the predictive roles of creative potential (DT fluency and originality) and creative personality (SRCT) on malevolent creativity, along with the moderating effect of morality. Age and gender were controlled in all analyses. In the first regression, fluency was the predictor, malevolent creativity the dependent variable, and morality the moderator. Originality and SRCT were the predictors in the second and third regression analyses, respectively. The interaction between morality and each predictor fell short of statistical significance; *F* (1, 208) = 1.19, *p* = 0.277, Δ*R*^2^ = 0.005 for creative potential, *F* (1, 210) = 0.01, *p* = 0.91, Δ*R*^2^ = 0.000 for fluency, and *F* (1, 210) = 0.01, *p* = 0.948, Δ*R*^2^ = 0.000 for originality (Table 3). 

Therefore, the interaction term was dropped, then a hierarchical regression analysis was performed. Malevolent creativity was the dependent variable, and age and gender were entered in Step 1; morality in Step 2; and creativity related variables, SRCT, fluency, and originality, were added in Step 3. The results indicated that Step 1 explained a significant amount of variance in the malevolent creativity (*R*^2^= 0.06, *F* (2, 211) = 7.11, *p* = 0.001), Step 2 was not significant (Δ*R*^2^ = 0.01, *R*^2^ = 0.07, *F* (1, 210) = 1.08, *p* = 0.299), and Step 3 was significant (*R*^2^ = 0.30, *F* (3, 207) = 23.30, *p* = 0.000) and explained an additional 24% of the variance. Gender, fluency, and originality emerged as significant predictors, with male participants having a higher number of malevolent creativity responses (*M* = 4.02, *SD* = 4.39) than females (*M* = 1.99, *SD* = 3.14).

## 4. Discussion

A growing body of research has suggested that there is a dark side to creativity (D. H. Cropley et al., 2008; Gino & Ariely, 2012; McLaren, 1993; Niepel et al., 2015; Walczyk et al., 2008). The present study focused specifically on the contextual influences on malevolent creativity to explore how ordinary people demonstrate atypical behaviors in certain socially oriented problems. The study aimed to document how individuals shift between positively and malevolently creative solutions for given constructed social problem tasks.

### 4.1. Correlation Between Morality, Malevolent Creativity, Creative Potential, and Creative Personality

The correlation analyses revealed a significant relationship between malevolent creativity and both originality and fluency, the primary creativity indices examined in this study. These findings align with existing literature (Beaussart et al., 2013; Dumas & Strickland, 2018; Gino & Ariely, 2012; Gutworth et al., 2016; Kapoor, 2015; Perchtold-Stefan et al., 2020), further supporting the association between creativity and malevolent creativity. Given that creativity involves unconventional thinking, with creative ideas and actions often deviating from social norms and expectations, it is unsurprising to observe this moderately positive relationship. Another potential explanation is that both malevolent creativity and originality focus on novel responses, as indicated by Harris et al. (2013). Thus, individuals who exhibit more malevolent creativity are likely capable of generating benevolent creative ideas when the social context no longer triggers aggressive responses. Conversely, someone with a plethora of original ideas is also likely to generate malevolently creative ideas.

The results showed no significant relationship between malevolent creativity and creative personality. Possessing traits conducive to creativity does not inherently lead to developing malevolent ideas. Instead, factors like intentionality, motivation, and goal setting might be more pivotal in determining whether one invests effort in generating benevolent or malevolent ideas and behaviors (A. J. Cropley, 2010; D. H. Cropley et al., 2008; Gruber, 1993; Runco, 2010). Individuals with a creative personality may consider various behavioral responses, including malevolent ones, when faced with a contextual stimulus, but they do not necessarily act in a malevolent manner. Kipper et al. (2010) found that creativity is not correlated with impulsivity; thus, creative individuals may choose to adhere to conventional ideas and behaviors when they are more suitable for the context (Runco, 2004).

Contrary to expectations, there was also no significant correlation between morality and malevolent creativity. This finding aligns with literature suggesting that creativity and morality are independent (Gruber, 1985; Niepel et al., 2015; Runco, 1993, 2009), yet it contradicts some aspects of malevolent creativity literature. One possible explanation is that current literature provides insights into specific aspects of morality, such as lying (Walczyk et al., 2008), hostility (Feist, 1998), and dishonesty (Beaussart et al., 2013). However, the broader relationship between malevolent creativity and morality remains underexplored and warrants further investigation. Additionally, the morality measure used in this study focused on proactive moral behaviors rather than on general moral values and reasoning, which may allow for the expression of both moral behaviors and malevolent creativity without a definitive moral stance. For example, Al Capone, one of history’s most notorious criminals, opened one of the first soup kitchens during the Great Depression. Thus, engaging in some moral behaviors provides limited insight into one’s moral stance. People may practice moral behaviors to enhance their self-image or gain social/self-rewards, even if their moral values do not restrict their personal beliefs, behaviors, and thoughts (Batson, 2011; Batson et al., 1997; Watson & Sheikh, 2008). Batson (2011) argues that moral hypocrisy—where actions serve self-interest while appearing moral—is more common than moral integrity, which involves adhering to moral principles. A comprehensive understanding of moral integrity, encompassing both values and behaviors, might offer a clearer picture of an individual’s moral position.

Regarding morality, it was positively related to creative personality and showed a significant but weak correlation with fluency. The results indicated no significant relationship between originality and morality. While morality enforces moral values and norms, creative behaviors and ideas often challenge conventional values and the status quo (Runco, 2014). Despite appearing contradictory, these findings are consistent with the perspective that morality and creativity can coexist (Runco, 1993, 2009; Runco & Nemiro, 2003), and creativity can be effectively applied in moral domains such as political activism, charity work, and social relationships. Shen et al. (2019) argued that morality benefits from creativity, as a moral agent with creativity-related traits, such as openness and tolerance for ambiguity, may also exhibit greater tolerance and openness to personal differences.

In terms of creative potential, specifically fluency and originality, ideation may occur independently of moral considerations (see Campbell, 1960). Moral concerns might encourage individuals to generate a wide array of ideas to develop solutions aligned with their moral stance (Shen et al., 2019). However, there was no significant correlation between originality and morality, suggesting that the role of morality in facilitating or inhibiting originality should be explored further. This study provided only a limited contribution to the understanding of the association between creativity and morality, highlighting the need for additional research in this area.

### 4.2. Comparison of Malevolent Creativity in Different Social Context Scenarios

One of the study’s objectives was to examine how social context influences malevolent creativity. Participants were presented with three different socially constructed problems for each group of disturbing and non-disturbing Divergent Thinking (DT) tasks. The disturbing RPP DT tasks, unlike the non-disturbing SG tasks, included elements of unfairness, disturbance, and potential social and personal threats. Conversely, the non-disturbing SG tasks were based on everyday life experiences and did not inherently require malevolent creativity. The results from the paired *t*-test revealed that participants exhibited significantly more malevolent behaviors during the disturbing RPP tasks compared to the SG tasks. These findings align with previous research (Clark & James, 1999; Harris et al., 2013; Kapoor & Khan, 2019), which suggests that malevolent creativity varies significantly depending on the nature of the socially oriented problems and factors such as perceived threat, unfairness, and upsetting experiences.

The results supported the social information processing theory (Salancik & Pfeffer, 1978), which posits that social context provides cues for meaning-making and influences personal behaviors. We found a significant shift in participants’ creative expression in response to different contexts. Participants predominantly employed benevolent creativity when addressing non-disturbing social context problems. However, they tended to transition from benevolent to malevolent creativity as the social context’s tone and characteristics shifted. Notably, the study’s sample comprised undergraduate students from a prestigious university, underscoring that even individuals perceived as “ordinary” can express malevolent creativity when provoked by a social context that elicits malevolent responses or when subjected to unfair behaviors by peers or associates. This highlights the powerful impact of social context in shaping creative expression, demonstrating how environmental factors can lead individuals to adapt their creative strategies, sometimes in unexpected ways.

The results underscore that, while personality plays a significant role in the dark side of creativity (Harris et al., 2013; Lee & Dow, 2011), malevolent creativity often arises from the contextual characteristics individuals encounter. These contexts significantly influence personal motivation, intention, and goal setting (Runco, 2010). When faced with disturbing social scenarios, individuals can exercise discretion and consciously choose to express their aggression and anger (Runco, 1996).

ANOVA analyses elucidated the variations between individual problem tasks. All three disturbing RPP tasks prompted greater malevolent creativity compared to the non-disturbing SG Performance and Dress tasks. Interestingly, the Taste task did not show a significant difference from the disturbing tasks, yet it elicited more malevolently creative responses than the other non-disturbing SG tasks. This suggests that even seemingly neutral contextual problems can unexpectedly trigger malevolent creativity.

The sample consisted predominantly of undergraduate and graduate students, which might explain why the Taste task’s nature made it easier for them to exhibit physically and emotionally malevolent behaviors toward friends or classmates. Experiencing a similar situation with parents, co-workers, or bosses could yield significantly different outcomes, underscoring the critical role of social contexts in shaping responses and understanding the mechanisms behind malevolent creativity. This highlights the nuanced interplay between individual and contextual factors, emphasizing the need for a deeper exploration of how different environments influence the expression of creative behaviors.

Although the three disturbing DT tasks showed no significant differences in eliciting malevolent creativity, the Manager tasks stood out with the highest scores. This task was not only personally disturbing but also carried a social threat, suggesting that the manager’s actions could jeopardize the participants’ job security. These findings align with Baas et al.’s (2019) research, which indicates that high social threat can enhance malevolent creativity.

The mean comparison findings of this study represent a significant and unique contribution, opening new pathways to document how social context influences malevolent creativity. They also aid in identifying and assessing the factors that sway individuals’ mindsets or decisions to engage in malevolent versus benevolent creativity when faced with socially challenging tasks. This research underscores the intricate relationship between social threats and creative expression, paving the way for further exploration into how environmental pressures shape creative responses.

### 4.3. Predicting Malevolent Creativity

The investigation into whether morality moderates the relationship between malevolent creativity and both creative potential and creative personality yielded no supporting evidence. Correlation analyses revealed that morality and malevolent creativity were unrelated, and morality showed only a slight correlation with fluency, making the lack of a significant moderation effect unsurprising. This may be due to the flexible moral compass often found in creative individuals, allowing them to justify both positive and malevolent forms of creativity as necessary (Gino & Ariely, 2012).

The non-significant correlations might also stem from how morality was assessed. The CAAC-morality domain measure focuses on specific moral behaviors, potentially failing to capture an individual’s true moral values. Furthermore, the morality measure being self-reported adds another layer of complexity. A recent meta-analysis on creativity and unethicality highlighted significant discrepancies between self-report and more objective assessments of unethicality, which closely relates to morality. Notably, self-report measures showed no significant correlation between unethicality and creativity, whereas objective measures, such as behavioral observations, revealed a significant correlation (Storme et al., 2020). This suggests that more robust, objective measures may be necessary to accurately assess the interplay between morality and malevolent creativity.

After dropping the interaction term, a multiple hierarchical regression analysis was conducted, revealing that creative potential, fluency, and originality were positively associated with malevolent creativity. Gender also emerged as a significant predictor, with males displaying higher levels of malevolent creativity compared to female participants, aligning with previous findings (Lee & Dow, 2011; Perchtold-Stefan et al., 2020).

The relationship between creativity and gender has shown mixed results in the literature. A meta-analysis examining personal factors and creativity suggested that women tend to exhibit slightly more creativity than men, and that an androgynous gender identity is significantly linked to creativity (Da Costa et al., 2015). However, in the realm of malevolent creativity, the opposite trend is anticipated, as men are often more aggressive under stress (Verona & Curtin, 2006) and have higher rates of criminal offending (Heidensohn, 1997). Dudek and Verreault’s (1989) research found no gender difference in creative performance using the Torrance Tests of Creative Thinking (TTCT), yet boys were more likely to generate aggressive responses than girls. Bennett et al. (2005) noted that differences in female neurophysiology and the early development of social cognitive skills might explain why males are more prone to violence.

It is important to interpret these findings cautiously, as the malevolent creativity mean was nearly double for men, but the number of male participants was relatively small. It should also be noted that the measures employed in our study primarily facilitate reactive responses and ideation in response to existing stimuli or threats. Consequently, it is not possible to conclude that men are inherently more malevolently creative or more inclined to proactively seek malevolent innovations than women. Future research with a gender-balanced sample could offer deeper insights into the relationship between gender and malevolent creativity, potentially refining our understanding of how gender influences the expression of creativity in socially challenging contexts.

### 4.4. Limitations and Future Research

This study presents several limitations that warrant consideration. Firstly, the sample primarily comprised graduate and undergraduate students, whose perceptions and reactions to socially oriented problems may differ significantly from those of individuals in organizational settings, middle-aged adults, or the elderly. This demographic skew could limit the generalizability of the findings to broader populations.

Another limitation lies in the reliance on self-reported data. Employing more objective and comprehensive measures that encompass both moral behaviors and values, especially in the assessment of morality, could yield fresh insights into the relationship between malevolent creativity and morality. It is important to acknowledge that, given the documented positive correlation between creativity and deception (Walczyk et al., 2008), highly creative participants might not provide entirely honest responses on self-reported morality measures, even when their anonymity is assured. Regarding the self-report nature of some of the measures, it is also important to highlight that the significant correlations observed among the measures, such as morality and creative personality, alongside the weak and non-significant correlations between morality and performance-based variables, suggest a potential Common Method Variance (CMV) concern. As highlighted in a meta-analysis by Lebuda et al. (2021), the nature of assessment methods (self-report vs. performance based) may substantially influence research outcomes related to the interplay between creativity and personality, and it might be the case for the current research study utilizing self-report measures. Consequently, future research should incorporate diverse assessment methods to mitigate potential CMV effects

One limitation of our study is the order in which the measures were administered. Another measure was used between RPP and SG, but still the sequential presentation of measures may have influenced participants’ responses due to potential priming effects or response biases. Future research should consider not just diverse assessments but, when possible, counterbalance the order of measures through different survey platforms that allows a random order of measures. Recall also what was said about scoring the tests. It might be good to bring in a larger sample of raters and ensure that they are entirely blind regarding all assessment details.

It is important to acknowledge certain limitations related to the conceptual frameworks of malevolent creativity. Malevolent creativity can manifest in both reactive and proactive forms, yet the current study and its methodologies primarily focused on the former. Reactive malevolent creativity is characterized by the generation of harmful ideas or solutions in response to existing problems or stimuli, often leveraging creative ideas and behaviors in reaction to external factors in response to immediate threats or challenges. In contrast, proactive malevolent creativity involves the intentional and anticipatory development of harmful innovations, strategically identifying and exploiting potential weaknesses or opportunities for malevolence without any provocation. Future research could use this distinction with specific assessments and investigate their relationship with various dimensions of creativity, including ideation, originality, morality, and creative personality.

Furthermore, studying malevolent creativity in real-world contexts involves numerous legal and ethical challenges. As in many malevolent creativity studies (Clark & James, 1999; Harris et al., 2013; Walczyk et al., 2008), this research relied on hypothetical social context scenarios to measure malevolent creativity. While ideation is a potential predictor of actual behavior, participants in hypothetical scenarios do not face the real consequences of their ideas or need to exercise caution in expressing malevolent responses.

Thus, future research should strive to bridge the gap between hypothetical and real-life scenarios, exploring how the expression of malevolent creativity might differ in authentic settings. This could involve developing innovative methodologies that ethically capture real-world responses, enhancing our understanding of how malevolent creativity manifests across diverse contexts and populations.

### 4.5. Implications

This study opens exciting new avenues for future research. Firstly, our findings reveal that contextual characteristics significantly influence malevolent creativity. This suggests that malevolent creativity is not restricted to individuals with inherently criminal or malevolent tendencies. Instead, it indicates that everyone might harbor a creative dark side that surfaces in specific social contexts. While this study sheds light on how social contexts influence malevolent creativity, there remains a need for further exploration into the roles of personality, social contexts, and their interplay in driving individuals to channel their creativity towards undesirable outcomes.

In alignment with the design of this study, future research could address the current limitations in assessing morality by incorporating measures that capture not just moral behaviors but also moral values and beliefs. Secondly, while this study uniquely contributes to understanding the social context and the dark side of creativity, it did not extensively examine personality factors beyond creative personality. Future studies could delve into various personality moderators, such as the Big Five traits, religiosity, or flexibility. This would help explain why some individuals are more prone to malevolent creativity in certain social settings, whereas others consistently refrain from such behavior.

Additionally, future research could investigate the interaction between situational factors and personality variables, such as mood, mental health, and emotional control. This would help identify how personality factors contribute to the shift from positive to malevolent creativity, alongside social context variations. Finally, studies with more diverse samples or cross-cultural research designs could offer valuable insights into how these results differ across cultures or within a more varied general population.

This study highlights the profound impact of social context on malevolent creativity. It reveals a clear variation in how malevolent creativity is expressed, suggesting that experiences of injustice, social threats, and unsettling situations can prompt individuals to generate a diverse array of malevolently creative ideas. An additional significant contribution of the study is its support for the link between creative potential and malevolent creativity. It further corroborates the finding that men are more likely to exhibit malevolent creativity.

## Figures and Tables

**Table 1:** Descriptive statistics of the variables and malevolent creativity scores.

Variables	N	Min	Max	*M*	*SD*
Morality	216	0.35	2.55	1.32	0.48
Creative Personality (SRCT)	214	1.42	4.58	3.00	0.60
SG—Taste	213	0	5	0.42	0.86
SG—Performance	213	0	8	0.16	0.69
SG—Dress	216	0	3	0.20	0.53
SG—Total	216	0	10	0.77	1.46
RPP—Sally	216	0	7	0.46	0.98
RPP—Classroom	214	0	6	0.51	0.98
RPP—Manager	217	0	8	0.65	1.14
RPP—Total	217	0	20	1.61	2.50
Fluency	217	5	96	39.55	15.15
Originality	216	0	30	7.16	4.89

*Note*: SRCT = Self Report of Creative Traits; SG = Social Games; RPP = Realistic Presented Problems.

**Table 2:** Bivariate correlations between the main variables.

	1	2	3	4	5
1. Morality	-				
2. Creative Personality (SRCT)	0.34 **	-			
3. Malevolent Creativity	0.09	0.01	-		
4. Fluency	0.16 *	0.06	0.42 **	-	
5. Originality	0.11	0.05	0.49 **	0.71 **	-

*Note*: SRCT = Self Report of Creative Traits. * *p* < 0.05. ** *p* < 0.01.

**Table 3:** Multiple hierarchical regression analyses predicting malevolent creativity.

Predictors		Model 1			Model 2			Model 3		
		*B*	SE	β	*B*	SE	β	*B*	SE	β
Constant		5.89	1.49		5.36	1.57		3.17	1.67	
Age		0.03	0.04	0.04	0.02	0.04	0.03	0	0.04	0
Gender		−2.23	0.6	−0.25 **	−2.21	0.6	−0.24 **	−2.07	0.53	−0.23 **
Morality					0.51	0.49	0.07	0.17	0.46	0.02
SRCT								−0.23	0.36	−0.04
Fluency								0.04	0.02	0.17 *
Originality								0.25	0.06	0.35 **
*R* ^2^	0.06				0.07			0.3		
∆*R*^2^	0.06				0.01			0.24		
F	7.11 **				1.08			23.30 **		

*Note*: SRCT = Self-Report of Creative Traits. * *p* < 0.05. ** *p* < 0.01.

## Data Availability

The data that support the findings of this study are available from the corresponding author upon reasonable request.
